# Hydrogen-Induced Degradation of Metallic Materials—A Short Review

**DOI:** 10.3390/ma18030597

**Published:** 2025-01-28

**Authors:** Alicja Krella

**Affiliations:** Department of Erosion Processes, Institute of Fluid-Flow Machinery Polish Academy of Sciences, Fiszera 14, 80-231 Gdansk, Poland; akr@imp.gda.pl

**Keywords:** hydrogen embrittlement, diffusion coefficient, dislocation density, mechanical properties, steel, aluminium alloy, nickel alloy, titanium alloy

## Abstract

Hydrogen is currently used as an energy source, and there are already vehicles (cars and buses) that run on hydrogen in our public spaces. Additionally, in the chemical, petrochemical and nuclear industries, many devices come into contact with hydrogen. This short review covers a broad range of topics related to HE, i.e., the main hydrogen charging methods, aspects related to hydrogen diffusion in metallic materials, and the main models of hydrogen-induced material degradation and their assumptions, and discusses the influence of hydrogen on the properties and degradation of different metallic materials used in the industry based on the latest research results. This review focuses on the four primary groups of materials used in hydrogen devices, i.e., steels, aluminium alloys, nickel alloys and titanium alloys. The basic information on the influence of hydrogen on the structure and properties, mainly elongation, hardness and tensile strength, of these metallic material groups are presented. The influence of the method of hydrogen charging and the time of hydrogen saturation, as well as the effect of structure, on the content of hydrogen in the material, as well as on such material properties as hardness, tensile strength, and fatigue strength, is shown.

## 1. Introduction

The problem of protecting the environment and preventing global warming is very topical. It involves the elimination or reduction of carbon dioxide (CO_2_) released into the atmosphere. According to the European Commission, reducing CO_2_ emissions and producing clean energy are currently of the highest priority. The EEA report states that most CO_2_ emissions derive from road transport, which is responsible for a quarter of the EU’s greenhouse gas emissions [1]. In this context, eliminating or reducing CO_2_ emissions from road vehicles has the greatest impact. The recommended means of road transport are electric vehicles or vehicles that use hydrogen as a fuel. The product of hydrogen combustion is water, which makes driving such vehicles environmentally friendly. From this point of view, the use of hydrogen is ecologically beneficial and is therefore considered to be the best form of energy for the 21st century. However, unlike natural gases such as methane, hydrogen does not exist in its pure form in nature, so it has to be produced. Because hydrogen has less energy per volume than natural gases (methane, methanol, propane, or octane, which represents gasoline) [2], it must be compressed. Furthermore, the volumetric energy density of any natural gas at any pressure exceeds the volumetric energy density of hydrogen. For example, the volumetric energy density of methane exceeds the volumetric energy density of hydrogen by about 3.2 times [2,3]. At a pressure of 800 bar, gaseous hydrogen reaches the volumetric energy density of liquid hydrogen. However, hydrogen in the liquid state has to be stored in high-tech high-pressure tanks or cryogenic containers. This means that hydrogen requires a completely new distribution network. From an economic point of view, this means that the cost of hydrogen production should be as low as possible. Unfortunately, the hydrogen production process involves energy losses. In addition, the liquefaction of hydrogen requires a large amount of energy. On the other hand, as has happened in the past, it can be expected that the development of a production technology will contribute to a reduction in costs. According to [4], the cost of hydrogen production and transport is expected to fall from $22.7 per kg H_2_ to $14.7 per kg H_2_ with an assumed increase in market share from 0.1% to 1%. In addition, the 2050 climate neutrality agenda of the Paris Agreement, the European Green Deal and the Fit for 55 package have contributed toward the energy transition and to work on the development of the hydrogen economy, which will require market development related to demand growth and people’s access to hydrogen.

Hydrogen-powered vehicles (cars and buses) are currently in public use. This means that the high-pressure hydrogen tanks, valves, pressure sensors, hydrogen accumulators, pipelines, etc. are exposed to hydrogen. As climate change and pollution become increasingly problematic, the use of hydrogen is expected to increase, particularly in the transport sector, as well as in the nuclear, chemical and petrochemical industries. As a result, the number of structures and components used for hydrogen production, storage and transportation that are thus exposed to hydrogen gas will increase. The first hydrogen-induced failures of steel tanks occurred in the 1960s [5]. Despite many studies, anti-destruction efforts and existing applications, the problem of hydrogen-induced damage remains. Shirband et al. [6] have reported that 25% of equipment failures in the petroleum industry are hydrogen induced. Hydrogen-induced fractures are also one of the limiting operational criteria for nuclear fuel safety in the nuclear energy sector [7,8]. This shows that the problem of hydrogen-induced fracture is important and requires a thorough understanding of the problem and the development of methods to eliminate or reduce it.

Due to the small size of the hydrogen atom, it can be absorbed by most metallic materials, affecting their strength properties and service life. The main effect of hydrogen on the material is a reduction in plasticity and accelerated cracking, which is known as hydrogen embrittlement (HE). The consequence of HE is a reduction in ductility (elongation in tensile tests can be as much as 50% for steels [9,10] or as much as 87% for nickel-based alloys [11]). Hydrogen also reduces tensile strength in many metallic materials [12,13,14] and the fatigue limit [15,16,17]. This is mainly due to an increase in the crack growth rate. The extent of the reduction in mechanical and strength properties depends on the material tested, the hydrogen content and the test conditions [14,15].

The subject of hydrogen-induced degradation is very broad and many reviews have been published [6,18,19,20,21,22,23,24,25]. For this reason, these reviews have presented the problem in a narrow context. Refs. [6,18,19,23,25] presented the HE problem only in relation to steel, starting from the presentation of different types of hydrogen damage in steel [6] to the presentation of different mitigation methods [18]. The main objective of [20] was to present models of HE mechanisms and the prevention of this type of damage by cadmium and nickel plating. The HE problem in high-pressure hydrogen tanks made of aluminium alloys is presented in [21]. Due to the number of studies and issues related to HE, it is not possible to cover everything in one review. It is even less possible to present and discuss all aspects of HE in a short review. Therefore, the aim of this review is to briefly present the main aspects of HE and the degradation of metallic materials used in industry.

Because investigations of the effect of hydrogen on mechanical properties and damage growth are impossible without hydrogen entering into the material structure, the author considered it important to begin with a presentation of the main hydrogen charging methods. The next important issue for understanding the problem is to know the aspects related to hydrogen diffusion in metallic materials and damage mechanisms. Finally, the effects of hydrogen on the properties of four groups of materials, such as steels, aluminium alloys, nickel alloys and titanium alloys, used for components exposed to direct contact with hydrogen, are presented, rather than just one group as in previous reviews. This means that this short review, although concise, covers a wider range of topics than previous reviews and is based on the latest research findings.

## 2. Hydrogen Charging

In order to study the effect of hydrogen on material properties, it must be introduced into the material structure. The two main methods used for hydrogen loading are the electrochemical loading process and high-pressure hydrogen gas loading.

**Figure 1:**
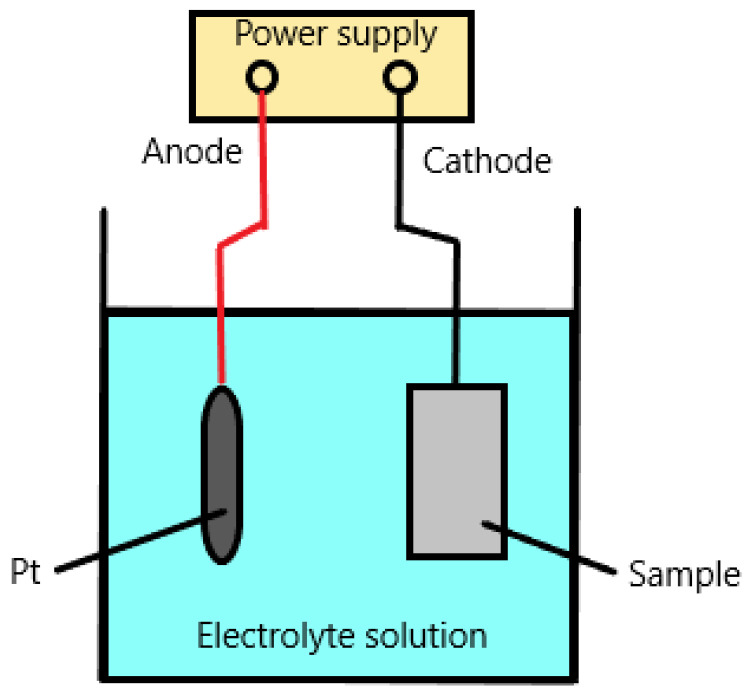
Schematic of set-up for cathodic hydrogen charging.

The electrochemical charging method is shown in Figure 1. In this method, the tested material is the cathode and the platinum electrode is the anode. However, other materials, such as graphite, can also be used as anodes [26]. Due to the simplicity of this setup, this method is widely used.

The amount of hydrogen that enters the material depends on the electrolyte solution used as the hydrogen source, the current density and the charging times. The most commonly used electrolytes are sulphuric acid, although other electrolytes are also used, such as NaCl, NaOH and H_3_PO_4_. To prevent the formation of hydrogen molecules (H_2_) and to improve hydrogen absorption, a poison is added. The poisons used are sulphur, arsenic and thiourea. The current density in the cathodic charging depends on the material being tested and the aim of charging. In [20], it was shown that the current density for hydrogen entry into a steel structure is between 0.02 mA cm^−2^ and 40 mA cm^−2^. Similarly, charging times are also in a wide range from one minute [27] up to 120 h [28]. However, the most common charging time was 24 h [28,29,30,31,32]. Some examples of the solutions and current densities used in cathodic hydrogen charging are shown in Table 1. In most cases, cathodic charging was carried out at room temperature. Nevertheless, higher temperatures are also used. Lu et al. [11] performed cathodic hydrogen charging of Ni-based superalloys at 75 °C. Pałgan et al. [30] performed hydrogen charging at current densities of 1, 10, 20 and 100 mA cm^−2^, charging times of 24, 48 and 72 h, and temperatures of 25 °C, 50 °C and 80 °C to investigate the effect on hydrogen content in the tested material (A516 carbon steel, 304L and 316L stainless steels). The greatest increase in the hydrogen uptake was seen when the current density increased from 0 to 20 mA cm^−2^, irrespective of the steel tested. The effect of a further increase in the current density value depended on the steel tested, with the hydrogen uptake decreasing slightly in A516, while increasing slightly in both stainless steels. Although the increase in charging time and temperature increased the hydrogen uptake for all materials tested, the extent of this increase depended on the material tested.

In the high-pressure hydrogen gas exposure method, hydrogen is charged using an autoclave [38,39]. Hydrogen charging parameters are gas pressure, temperature and time. A detailed analysis of the results presented in [38,39] shows that, in order to obtain a similar degree of hydrogen content in the material and with the same saturation time, it is necessary to use a higher process temperature while reducing the pressure. While maintaining the same hydrogen pressure in the autoclave, the charging time should be shortened with the increase of the process temperature. Nevertheless, regardless of the pressure and temperature used, the charging time is much longer than for the cathodic charging method. Examples of charging conditions are as follows: the hydrogen pressure of 10 MPa, temperature of 400 °C, and charging time of 200 h [38], or the hydrogen pressure of 120 MPa, temperature of 280 °C, and charging time of 400 h [39].

The next method of hydrogen charging is the thermodiffusion method, which is carried out in a furnace at high temperatures [40,41,42,43]. In this method, the material is placed in a furnace, the pressure in the furnace is reduced to approximately 5 × 10^−4^ Pa, the temperature is then raised and hydrogen is added to the furnace. Depending on the alloy tested, the temperature ranges from 600 °C to 1000 °C [40,41,42,43]. For example, in [40], which tested a titanium alloy with a near α-phase structure, the charging temperature was 750 °C, the hydrogen flow rate was 1 L min^−1^, and the charging time was adjusted to obtain the desired hydrogen content. The material was then held at 750 °C for 1 h to allow hydrogen to penetrate and finally, the material was air cooled to room temperature. In [43], an α + β phase Ti-6Al-4V alloy was charged with hydrogen at 800 °C for 3 h.

**Figure 2:**
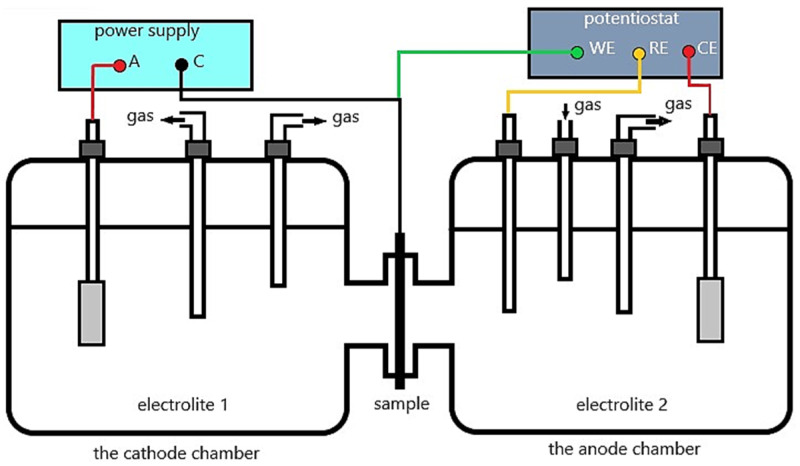
Schematic of the Devanathan–Stachurski electrochemical cell, A—anode, C-cathode, WE—working electrode, RE—reference electrode, CE—counter electrode, gas—argon, N_2_, H_2_S, etc.

In addition to these methods of charging materials with hydrogen, hydrogen permeation is also being investigated. For this purpose, the Devanathan–Stachurski electrochemical cell is used. This method is used to measure the hydrogen diffusion coefficient, D, and the hydrogen concentration, C. The Devanathan–Stachurski cell is a double electrochemical cell (Figure 2). The tested sample, which is the membrane, is placed between the two electrolytic cells. Hydrogen atoms are generated on one side (the entry side) of the membrane (tested material) and the diffusing hydrogen atoms are oxidised on the other side (the exit side). Aqueous solutions of NaOH or H_2_SO_4_ are usually used on the entry side, and an aqueous solution of NaOH is the typical solution on the exit side. Electrochemical charging is more commonly used at temperatures up to 100 °C [23].

The hydrogen content in the material structure is measured by thermal desorption spectroscopy (TDS) [14,15,44], hydrogen microprint technique (HMPT) [45], secondary ion mass spectrometry (SIMS) [46] and scanning Kelvin probe force microscopy (SKPFM) [47].

## 3. Diffusion of Hydrogen

The very small size of atomic hydrogen (31 ± 5 pm/0.03 Å) allows it to penetrate relatively easily into the structure of materials through free surfaces and the first few atomic layers below surfaces; grain boundaries (GBs), especially high-angle grain boundaries (HAGBs); dislocations; vacancies/Schottky defects; triple junctions (TJs); stacking faults; inclusions; precipitate/matrix interfaces; and strain fields around precipitates and voids in the crystallographic lattice. The size of the voids depends on the crystallographic structure of the material and the size of the atoms of the elements that make up the material. Metallic materials have three main crystallographic lattices: regular body-centred-cubic (BCC), regular face-centred-cubic (FCC) and hexagonal close-packed (HCP). In the BCC materials (e.g., pure iron, ferritic steel), voids in the crystallographic lattice can be filled by an atom with a radius 0.291 × R (the radius of the atoms making up the lattice). In FCC materials (e.g., austenitic steel, aluminium, copper, nickel and their alloys), there are six larger octahedral voids, which can be filled with an atom with a radius of 0.414 × R and eight smaller tetrahedral voids, which can be filled by an atom with a radius of 0.255 × R. HCP materials (e.g., titanium, magnesium and their alloys) have the same size of voids.

In the case of steel, the main element is iron, the atomic radius of which is 126 pm. This causes the voids in BCC steels (e.g., carbon and ferritic steels) to have a size of 36.6 pm, while in FCC steels (e.g., austenitic steels) the voids have a size of 52.16 pm and 32.13 pm for octahedral and tetrahedral voids, respectively. However, alloying elements, such as chromium and nickel, have a radius of 128 pm and 124 pm, respectively, so they occupy the position of iron in the crystallographic lattice, which slightly reduces the void size. Some voids in steel can be filled by carbon atoms, which have an atomic radius of 70 (67) pm. This causes the deformation of the crystal lattice and is also the reason for the very low solubility of carbon in α-iron. As a result, only a part of the voids can be filled by hydrogen. Considering the larger size of voids in the FCC lattice, the hydrogen solubility in FCC steels is also larger than that in BCC steels. In the case of aluminium, which also has an FCC crystal structure, while its atomic radius is 143 pm, the voids have respective sizes of 59.2 pm and 39.46 pm. Given the size of the hydrogen atoms, the solubility of hydrogen and HE is likely to be greater than in BCC steels.

In contrast to atomic hydrogen, molecular hydrogen H_2_, which has a size of 2.89 Å [48], is too large to enter the crystallographic lattice. Therefore, hydrogen diffusion mainly concerns atomic hydrogen.

The introduction of hydrogen into the material structure obeys the diffusion law, which is described as follows:

(1) $$D=D_{0}exp\left(-Q/RT\right)$$

where D_0_—diffusion coefficient, Q—activation energy for diffusion, R—gas constant, and T—temperature in K.

The diffusion coefficient is a material property and, similar to other properties, such as hardness, it depends on the material structure. According to Equation (1), diffusion depends on the temperature. In addition, the diffusion coefficient, D_0_, also depends on the test temperature and the crystallographic lattice [5,20,24,49]. Lynch [5] showed that the diffusion coefficient of BCC materials (iron) is higher than that of FCC and HCP materials (e.g., nickel and titanium, respectively) at temperatures below 600 °C. Above this temperature the relationship is reversed. Moreover, the effect of temperature on the diffusion coefficient of BCC materials is much less than of FCC and HCP materials. According to Nelson [50], the lattice hydrogen diffusion coefficient for α-ferrite is D_0_ = 2.3 × 10^−7^ m^2^ s^−1^ at a temperature of about 150 °C. Tolstolutska et al. [51] showed that the diffusion coefficient of iron is larger and equals 6.7 × 10^−7^ m^2^ s^−1^, with a temperature range of 100 °C to 500 °C. On the other hand, Li et al. [24] showed that the hydrogen diffusion coefficient for pure iron is much lower and equals 5.8 × 10^−10^ m^2^ s^−1^. Considering that diffusion goes through grain boundaries, the grain size is expected to have a significant effect on the diffusion coefficient, as in the case of hardness. However, Park et al. [28] have showed that the grain size has little effect on the diffusion rate. Despite many studies, the reason for the differences in the hydrogen diffusion coefficient values has not been explained.

In the case of multiphase materials, the structure plays a key role in the diffusion coefficient. For example, SAE1008 steel, which contains ferrite and carbides, has a diffusion coefficient of 2.19 × 10^−10^ m^2^ s^−1^, while the diffusion coefficient of SAF2205 steel, which has ferrite and austenite grains, is 3.0 × 10^−15^ m^2^ s^−1^ [24]. The difference in coefficient value is therefore three orders of magnitude. The same work reports that PSB1080 steel, which is composed of martensite and bainite, has a hydrogen diffusion coefficient of 4.43 × 10^−11^ m^2^ s^−1^. On the other hand, and as shown in [52], the content of each microstructural fraction also affects the coefficient. The diffusion coefficient of the martensitic steel, which was composed of 23% of martensite and 77% of a lower bainite fraction, was 3.71 × 10^−11^ m^2^ s^−1^. When the martensite fraction increased to 45.8% and the lower bainite fraction decreased to 44.2%, the diffusion coefficient increased to 5.13 × 10^−11^ m^2^ s^−1^.

## 4. Degradation Mechanisms

Despite many studies, there is not one theory describing the mechanism of degradation caused by hydrogen, but several, as follows:

(1) Hydrogen-enhanced decohesion mechanism (HEDE) [5,18,20,53,54];
(2) Hydrogen-enhanced local plasticity model (HELP) [5,18,20,54,55];
(3) Adsorption-induced dislocation emission (AIDE) [5,20,56];
(4) Hydrogen enhanced macroscopic ductility (HEMP) [20,21];
(5) Hydrogen changed micro-fracture mode (HAM) [20];
(6) Decohesive hydrogen fracture (DHF) [20,21];
(7) Mixed fracture (MF) [20];
(8) Hydrogen-induced phase transformation [18];
(9) Hydrogen assisted micro void coalescence (HDMC) [20,57].

However, the most commonly analysed models by researchers are the hydrogen-Enhanced decohesion mechanism (HEDE), the hydrogen-enhanced localised plasticity (HELP) and the adsorption-induced dislocation emission (AIDE) models. These three basic models of hydrogen embrittlement mechanisms are presented below.

**Hydrogen-Enhanced Decohesion Mechanism** (HEDE) [18,20,53,54].

**Figure 3:**
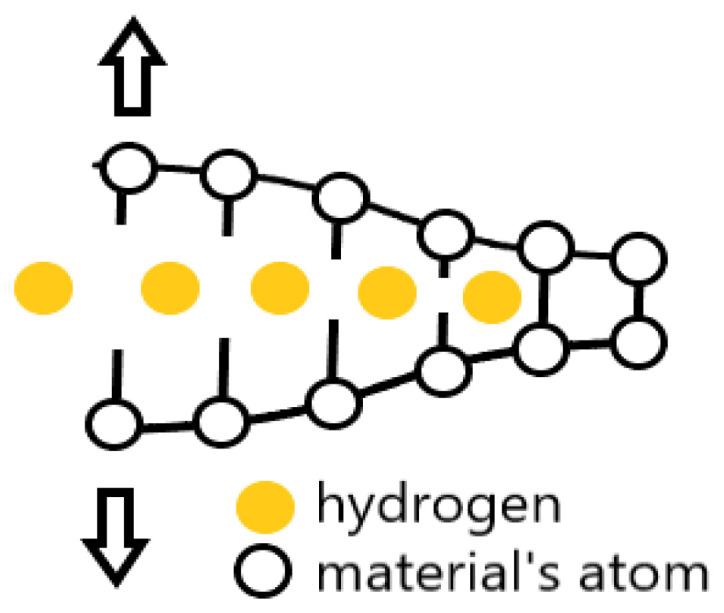
HEDE mechanism—broken interatomic bonds under tensile stress.

This mechanism was the first proposed mechanism and is the simplest of the models. It is based on the idea of reducing the cohesive strength of the material at the crack tip with the hydrogen atom (Figure 3). Under loading conditions (tensile stress), the hydrogen atoms reduce the interatomic or cohesive strength of the material at the crack tip, leading to the formation of a cleavage-like crack. As a result of the reduction in the cohesive strength of the material, the surface energy is reduced, in turn reducing the fracture stress and causing the crack to occur below the allowable value. The HEDE mechanism is based on the fact that hydrogen atoms diffuse to high-stress sites, with the crack tip being such a site. In addition, the density of hydrogen traps increases along the crack path.

**Hydrogen-Enhanced Local Plasticity Mechanism** (HELP).

**Figure 4:**
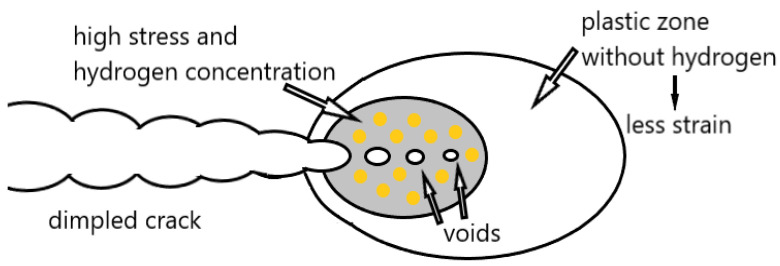
HELP mechanism.

This mechanism is a widely accepted mechanism of hydrogen embrittlement and is developed based on studies of the activation energy of dislocation motion in the presence of hydrogen and fractographic studies of metallic materials. Based on results that show that the activation energy for the dislocation motion is reduced by the hydrogen atom and that the activation region is reduced, it is assumed in this model that the hydrogen accumulated near the crack tip reduces the resistance to dislocation motion. The dislocation mobility increases and the dislocations behave as carriers of plastic deformation. As a result, local plastic deformation and slip bands can be generated even in a brittle material, but with a decrease in macroscopic ductility. Therefore, different fracture modes are observed in hydrogen-induced failure, such as intergranular, transgranular and quasi-cleavage. The fracture mode is influenced by hydrogen concentration, microstructure and crack tip stress intensity. The HELP mechanism is shown schematically in Figure 4.

**Adsorption-Induced Dislocation Emission** (AIDE).

**Figure 5:**
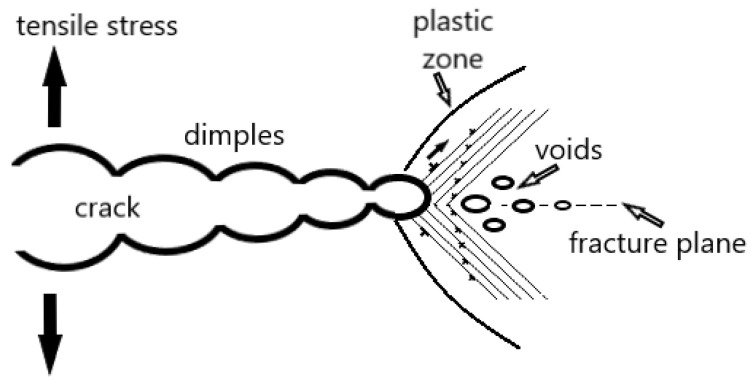
The AIDE mechanism.

This model is a combination of the HEDE and HELP models (Figure 5). It is assumed that dissolved hydrogen atoms are adsorbed at crack tips, i.e., in the stress concentration region. As a result, the interatomic bond or cohesive strength of the material is weak according to the HEDE mechanism. The dislocation movement is facilitated and leads to crack growth, which, according to the mechanism in the HELP model, occurs by slip and micro-void formation. Thus, in the AIDE model, crack nucleation and growth are the result of decohesion and dislocation emission at the crack tip. Crack growth and simultaneous cracking occur due to the combined effect of crack tip slip with microvoid coalescence.

## 5. Hydrogen’s Effect on Materials Properties

In many practical applications, mechanical and strength properties determine the service life of structural elements. As mentioned in the introduction, 25% of equipment failures in the oil industry are caused by hydrogen. For this reason, knowledge of the effect of hydrogen on their change is crucial for the proper planning of maintenance work. The effect of hydrogen on mechanical properties has been mainly investigated using the slow strain rate tensile (SSRT) test [10,11,14,16,28,29]. However, the hardness measurements [34], tension–compression fatigue tests [15,17,58], the Charpy test [35] and the scratch test to measure the adhesion of coatings exposed to hydrogen charging [33] have also been used. The effect of hydrogen on the degradation of different types of metallic materials is presented below.

### 5.1. Steel

Iron and its alloys contain large amounts of interstitial voids that can be occupied by hydrogen. The hydrogen found there is known as dissolved hydrogen. In addition, steels contain various defects beyond the dislocations that interact with dissolved hydrogen. Hydrogen in these defects is known as trapped hydrogen. Hydrogen entry can occur during steel fabrication, processing and service life. The extent of the effect of hydrogen uptake in steel on mechanical properties, i.e., surface hardness and reduction of elongation and tensile strength, is highly dependent on the chemical composition of the material, which determines the crystallographic lattice and the charging conditions. Iron, carbon steels and alloyed steels with ferritic and ferritic–perlitic structure have BCC crystallographic lattices, while austenitic steels have FCC lattices. The most commonly tested steels are austenitic steels, i.e., 316 and 304 steels. However, carbon steels and pipeline steels have also been investigated.

In the case of 316L steel, hydrogen charging performed in 0.5 mol L^−1^ H_2_SO_4_ with the addition of 1 g L^−1^ Na_3_PO_4_ · 12H_2_O as a poison and a current density of 20 mA cm^−2^ for 96 h at room temperature increased the surface hardness by 11.95%. SSRT testing conducted at a strain rate of 2.8 × 10^−4^ s^−1^ showed that tensile strength decreased by only 0.67%. However, the reduction in elongation was much greater (13.93%) [34]. When this steel was subjected to laser peening before hydrogen charging, the effect of hydrogen charging on surface hardness and elongation was weaker: surface hardness only increased by 1.18%, and elongation decreased by 9.66%; however, tensile strength decreased by 1.73%. The smaller increase in hardness due to hydrogen charging was associated with an increase in hardness after laser shot peening and a reduction in residual stress: from a tensile stress of 24 MPa to a compressive stress of −233 MPa. These investigations showed that the smaller the increase in surface hardness after hydrogen charging, the smaller the reduction in elongation. On the other hand, Hatano et al. [38] showed an increase in elongation (5.4%) of this steel (316) when tested in a hydrogen atmosphere and compared with the result obtained in vacuum.

The effect of hydrogen charging on the properties of 316L steel was also studied by Herms et al. [29]. They also used the SSRT tests, but the tests were performed at three strain rates: 7 × 10^−7^ s^−1^, 1 × 10^−6^ s^−1^ and 5 × 10^−6^ s^−1^. As the strain rate increased, the strain to fracture decreased, while the crack propagation rate increased. The strain to fracture decreased from 20% for the strain rate of 7 × 10^−7^ s^−1^ to 15%, for the strain rate of 5 × 10^−6^ s^−1^, whereas the crack propagation rate increased from 5 × 10^−10^ m s^−1^ to 28 × 10^−10^ m s^−1^, respectively. In the hydrogen penetration zone, i.e., at the sample’s surface, multiple brittle, transcrystalline and intercrystalline cracks were observed. In the deeper zone, a ductile crack appeared in the form of dimples.

**Figure 6:**
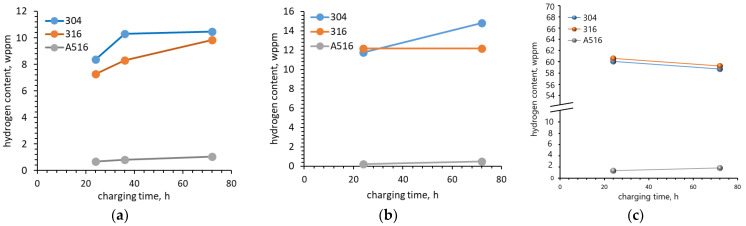
Total hydrogen content in the material introduced due to (**a**) cathodic charging at 20 mA cm^−2^ and 24 °C; (**b**) gaseous charging at 180 °C; (**c**) gaseous charging at 360 °C, based on [30].

Pałgan et al. [30] investigated the effect of current density, charging time and charging temperature on the hydrogen uptake in austenitic 316L and 304L steels and low alloyed A516 steel using cathodic and high-pressure gaseous hydrogen charging. Hydrogen uptake in austenitic steels was much higher (to the order of 7–8 weight parts per million (wppm)) than in low alloyed ferritic–perlitic steel (to the order of 0.7 wppm). These results contradict the hydrogen diffusion coefficients for BCC and FCC materials presented by Lynch [5], who showed that the hydrogen diffusion coefficients for BCC materials are several orders of magnitude greater than those for FCC materials at ambient temperatures. On the other hand, considering that austenitic steel has FCC structure and A516 steel has BCC structure, we can ascertain that the reason was the size of the crystallite voids. Increasing the current density from 20 mA cm^−2^ to 100 mA cm^−2^ increased the total hydrogen uptake in 304L steel from 8.36 wppm to 8.68 wppm (3.83%) (Figure 6a). In the case of 316L steel, the effect was much less and the total hydrogen uptake only increased from 7.28 wppm to 7.34 wppm (0.82%). In opposition to the total hydrogen uptake, the trapped hydrogen uptake decreased by 11.85% for 304L steel and 9.5% for 316L steel. In the case of A526 steel, the total hydrogen uptake decreased from 0.69 wppm to 0.66 wppm (4.35%) and the trapped hydrogen uptake also decreased. This reduction reached 15.4%. Charging time and electrolyte temperature had a much greater effect on the increase in the hydrogen uptake. As the charging time increased from 24 h to 72 h, the total hydrogen uptake increased by 25.1%, 35% and 53.6% for 304L, 316L and A516 steels, respectively. Although the greatest increase was observed for A516 steel, the total uptake for this steel was the lowest (only 1.06 wppm after 72 h of charging compared with 10.31 wppm for 304L steel). In the case of gaseous charging (Figure 6b,c), the difference in hydrogen uptake between the steels tested was even greater. For example, charging at 200 bar pressure and temperature of 180 °C in pure H_2_ for 72 h resulted in a total hydrogen uptake of 0.49 wppm for A516 steel compared with 14.81 wppm and 12.17 wppm for 304L and 316L, respectively (Figure 6b). When hydrogen charging was at 360 °C, the hydrogen uptake was 1.83 wppm, 58.73 wppm and 59.28 wppm, respectively. The results obtained by Pałgan et al. [30] show that gaseous hydrogen charging, especially at 360 °C, is much more effective compared with cathodic hydrogen charging (Figure 6c). One of the reasons may be the higher charging temperature. The lower hydrogen absorption in 304 steel shown in this study may indicate a higher resistance to HE. A study by Hatano et al. [38] contradicts this conclusion. When tested in a hydrogen atmosphere, they reported a reduction in elongation of up to 67% for 304 steel when compared with the sample tested in a vacuum atmosphere, while for 316 steel they reported an increase in elongation.

Regardless of the research into the influence of hydrogen charging conditions on hydrogen content in materials with different crystallographic lattices, another important question is how such a specific level of hydrogen content affects the strength properties. In other words, whether, for example, the content of 1 wppm of hydrogen in the BCC lattice causes a correspondingly smaller negative effect than 10 wppm of hydrogen in the FCC lattice. The effect of hydrogen content on HE was investigated by Takai and Watanuki [14] in a low-alloyed, high-strength structural steel, which was thermally treated to obtain different microstructures. Hydrogen was charged in an aqueous solution of 20 mass% NH_4_SCN at a temperature of 50 °C for 24 h. The hydrogen content was 3 wppm in the steel with a ferritic and bainitic structure. In the case of steel with a tempered martensite structure, the hydrogen content increased to 4 wppm. Compared with the results obtained by Pałgan et al. [30], who also tested low-alloyed steel, the content of hydrogen was much higher. The maximum stress and plastic elongation in the SSRT tests of steel with a ferrite and bainite structure decreased by 14% and 82%, respectively, with increasing immersion time, in contrast to the steel with tempered martensite structure, whose maximum stress and elongation did not change. This shows that the hydrogen content cannot be the only parameter indicating an expected reduction in elongation, but that the material structure must also be taken into account.

In the case of the X100 pipeline steel [10], which is a low-alloyed steel with a heat treatment-dependent structure, the tensile strength remained nearly unchanged in the hydrogen-charging SSRT tests at a strain rate of 1.12 × 10^−6^ s^−1^, irrespective of the heat treatment applied, while the elongation and reduction-in-area decreased. The extent of this decrease varied with the steel condition, which also affected the diffusible H content. The greatest reduction in elongation (from 38.8%) was achieved for the steel after hot rolling at 1100 °C with an average grain size of 28.7 μm. The steel in this condition had the highest concentration of diffusible H (0.46 ppm). Austenitisation at 1000 °C for 3 h and cooling in water at a cooling rate of 600 °C s^−1^ after the hot-rolling process resulted in a grain size of 34.6 um. Hydrogen charging resulted in a diffusible H content of 0.22 ppm and a reduction in elongation (33.8%). Cooling in air at a cooling rate of 20 °C s^−1^ after austenitisation resulted in a grain size of 30.6 μm, a diffusible H content of 0.19 ppm and a reduction in the elongation of 25.9%. Considering that the structure of this steel was the same after the hot-rolling process and after air cooling and consisted of ferrite, martensite and austenite, the grain size and especially the high-angle grain boundaries influenced the diffusible H content and the reduction in elongation.

**Figure 7:**
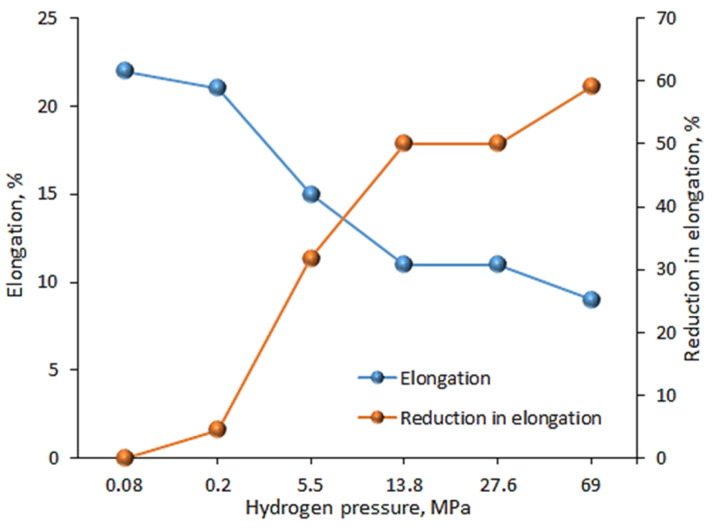
Correlation between the hydrogen gas pressure and elongation.

Martin et al. [25], who also tested X100 pipeline steel, showed that the reduction in elongation due to testing in a hydrogen atmosphere could be even greater (Figure 7). At a hydrogen pressure of 5.5 MPa during the tensile test, elongation decreased by 32% (from 22% for the test in air to 15% for the test in a hydrogen atmosphere). For tensile tests at 13.8 MPa hydrogen pressure, the elongation decreased by 50% (from 22% to 11%). However, when the hydrogen pressure was increased to 69 MPa, the elongation decreased by 59%.

### 5.2. Nickel Alloys

Nickel has a face-centred cubic (FCC) crystal lattice similar to austenitic steel but with a slightly higher density. Due to their good strength and corrosion properties, nickel alloys are used in the oil and gas industry, where they are exposed to high pressure, high temperature and highly aggressive environments, such as corrosive gases containing large amounts of H_2_S. Such environments cause hydrogen to enter structural elements. For this reason, the study of HE in nickel alloys is very important.

**Figure 8:**
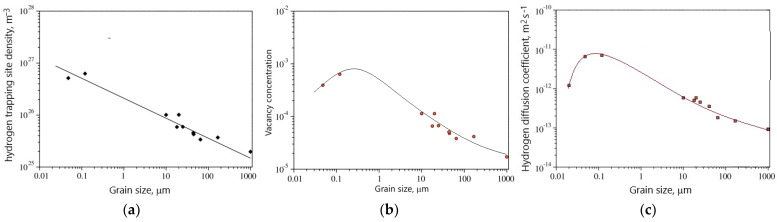
Correlation between grain size in pure nickel and (**a**) hydrogen trapping site density; (**b**) vacancy concentration; (**c**) effective hydrogen diffusion coefficient, based on [59].

Due to the numerous industrial applications of nickel alloys, where they are exposed to hydrogen, the first stage of HE research was to investigate the influence of structure, i.e., grain size, on hydrogen diffusion and its trapping sites. Oudriss et al. [59] studied the effect of grain size in pure nickel on hydrogen trapping sites and hydrogen diffusion. For this purpose, they used the technique of Devanathan and Stachurski. They first investigated the effect of grain size on the nature of the decrease of GB density. An increase in grain size from 20 nm to 168 μm resulted in an exponential decrease in GB densities. The electrochemical hydrogen permeation test showed that the density of hydrogen trap size, vacancy concentrations and diffusion coefficients were related to the grain size (Figure 8). With increasing grain size, i.e., decreasing GB density, the trapping site density decreased (Figure 8a). In the case of vacancy concentrations and diffusion coefficients, a threshold grain size was found at which they reached their maximum value (Figure 8b,c). Comparing the correlation between grain size and vacancy with the correlation between grain size and diffusion coefficient, a correlation between diffusion coefficient and vacancy density is seen, with the effective diffusion coefficient increasing with increasing vacancy density. They found that the acceleration of hydrogen diffusion along grain boundaries was mainly due to high-angle boundaries. The grain boundaries with low misorientation are preferential areas for hydrogen segregation. Moreover, hydrogen promoted the formation of vacancies around the GBs. The hydrogen diffusion coefficient values (Figure 8c) are much lower than those given by Lunch [5]. It can be expected that nickel alloys will have better resistance to HE than austenitic steels.

**Figure 9:**
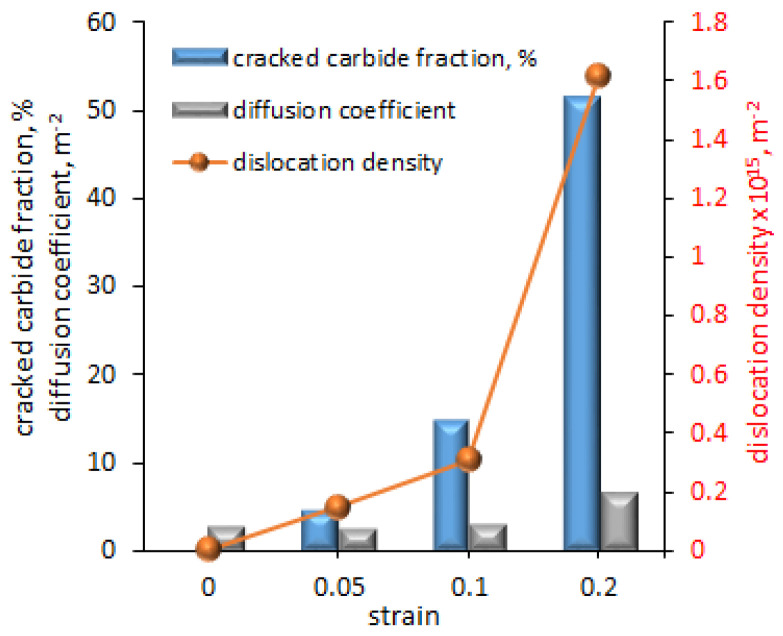
The effect of strain on cracked carbides fraction, dislocation density and diffusion coefficient in nickel 625 alloy, based on [60].

Because, in industrial applications, the material is subjected to stresses that can cause deformation, Lu et al. [60] investigated the effect of plastic deformation of the 625 alloy, i.e., strain, on the dislocation density and ultimately on the effective diffusion coefficients (D_eff_). The alloy was pre-strained to three strain levels: ε = 0.05, 0.1 and 0.2, and was then hydrogen charged using a Devanathan–Stachursky permeability cell. An amount of 0.1 M NaOH was used in the oxidation cell, while 0.2 g L^−1^ thiourea was added to the charging cell to promote hydrogen adsorption. The pre-straining caused cracking of the carbides which precipitated along the grain boundaries and increased the dislocation density, which ultimately increased the effective diffusion coefficient (Figure 9). At a strain of 0.05, 4.47% of the carbides were cracked and the dislocation density was 0.15 × 10^15^ m^−2^. The permeation test showed a D_eff_ of 2.93 m^2^ s^−1^. Increasing the strain to 0.1 increased the cracked carbides to 0.31, the dislocation density to 0.31 × 10^15^ m^−2^ and D_eff_ to 2.65 m^2^ s^−1^. Thus, a twofold increase in strain caused a twofold increase in dislocation density and a threefold increase in cracked carbides, but had little effect on D_eff_. A further twofold increase in strain to ε = 0.2 resulted in a fivefold increase in dislocation density, a threefold increase in cracked carbides and a twofold increase in diffusion coefficient.

The effect of hydrogen on mechanical properties has been investigated by Lu et al. [11]. They tested two Ni-based superalloys: 718 and 725, which differed mainly in the iron content: the 718 alloy has 19.14 wt.% Fe and the 725 has 10.1 wt.% Fe. Both alloys were heat treated: the 718 alloy was aged at 782 °C for 6.5 h followed by air cooling and the 725 alloy was aged at 732 °C for 8 h and 621 °C for 8 h followed by air cooling. Then, they were hydrogen charged in a mixture of glycerol and H_3_PO_4_ for 18 h at a cathodic current density of 15 mA cm^−2^ at 75 °C. Hydrogen desorption spectra for both alloys showed that hydrogen was trapped on precipitates, GBs, and δ phase (Ni_3_Nb). Ultimate tensile strength (UTS) in the SSRT tests conducted at a strain rate of 2 × 10^−5^ s^−1^ was reduced by 10% and 31% and total elongation was reduced by 66.1% and 87.3% for the 718 and 725 alloys, respectively, when compared with these alloys in hydrogen-free condition. The resulting reduction in elongation is much greater than that obtained when testing austenitic steels [34,38]. Lu et al. [11] found that local increases in stress and strain concentration at the intersections of dislocation slip bands attracted hydrogen to these sites and promoted the formation of dislocations and vacancies, which ultimately contributed to the formation of microvoids and was responsible for the primary transgranular cracks. The intergranular cracks in alloy 718 were attributed to the slip localisation at the triple junctions of GB, highly disoriented GB and δ-decorated GB. In the presence of hydrogen, the formation of microcracks along the disjoint interfaces between δ-precipitate and matrix was facilitated by the HEDE mechanism. As a result, the hydrogenated alloys exhibited mixed mode fracture: brittle and ductile, whereas the hydrogen-free alloys exhibited ductile mode fracture. The brittle fracture was in the form of river patterns and ridges. Intergranular cracking occurred along both GBs and phase boundaries, but the most brittle region was dominated by transgranular cracking.

**Figure 10:**
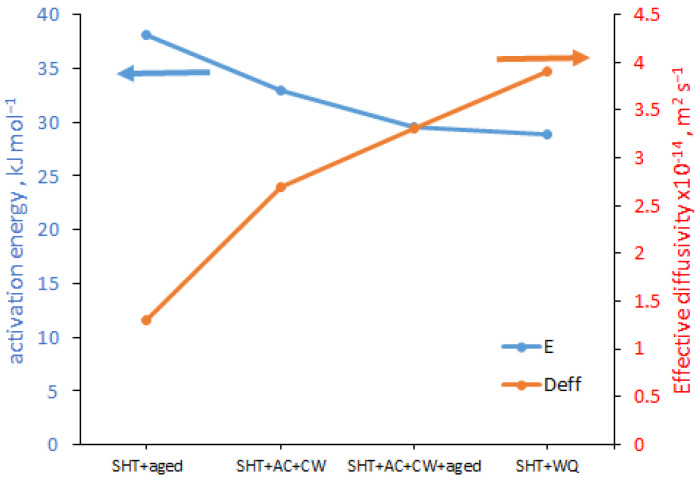
The activation energy and effective diffusion of Monel^®^ K-500, based on [61].

The nickel alloy most commonly used in the oil and gas industry, is Monel^®^. Monel^®^ K-500 is a nickel–copper alloy (64Ni–30Cu–3Al, wt.%) that is resistant to stress corrosion cracking (SCC) in various natural environments. In addition, an increase in the operating temperature causes a slight decrease in mechanical properties. For example, the yield strength of the alloy in the precipitation hardened condition decreases from 670 MPa to 570 MPa as the temperature increases from 20 °C to 500 °C. Ai et al. [61] investigated the effect of the isothermal treatment on hydrogen uptake and diffusivity. The alloy was solution heat treated (SHT) and aged (SHT + aged), SHT and air cooled (SHT + AC), SHT and AC and cold worked (SHT + AC + CW), SHT and AC and CW and aged (SHT + AC + CW + aged), and SHT and water quenched (SHT + WQ). The activation energy for H diffusion ranged from 28.9 ± 1.1 kJ mol^−1^ to 38.1 ± 3.6 kJ mol^−1^, and the effective H diffusivity at room temperature ranged from 0.9 m^2^ s^−1^ to 3.9 × 10^−14^ m^2^ s^−1^, depending on the alloy condition (Figure 10). Monel^®^ K-500 had the lowest activation energy and the highest effective diffusivity in the SHT + WQ condition, while the highest activation energy and the lowest effective diffusivity was found in the SHT + aged condition (28.9 + 1.1 kJ mol^−1^ and 3.9 × 10^−14^ m^2^ s^−1^ vs. 38.1 + 3.6 kJ mol^−1^ and 1.3 × 10^−14^ m^2^ s^−1^, respectively). They attributed the higher activation energy in the aged material compared with the solid solution condition to the transport barriers resulting from H trapping in the precipitates for SHT, SHT + aged, SHT + AC + CW and SHT + AC + CW + aged Monel^®^ K-500.

Harris and Burns [62] also investigated Monel^®^ K-500 after isothermal heat treatment, but they were interested in the susceptibility to cracking in a hydrogen environment. The alloy was solution treated at 950 °C for 1 h followed by water quenching and aged at 650 °C for 0, 0.5, 5 and 50 h, followed by water quenching. This heat treatment increased yield stress, tensile strength and Young’s modulus. However, the best mechanical properties were obtained after ageing for 5 h. The heat treatment had little effect on grain size and a fraction of Σ3 and Σ9 grain boundaries, but the radius of precipitations increased from 3.34 nm to 11.22 nm with increasing ageing time from 0.5 h to 50 h. The susceptibility to cracking was investigated in four environments: dry N_2_ gas and full immersion in aqueous 0.6 M NaCl electrolyte at applied potentials of −1000, −1100, and −1200 mV_SCE_. The crack growth rate of the unaged alloy increased almost linearly on a logarithmic scale with U+higher crack growth rate was obtained for the higher potential (−1100 mV_SCE_). In the case of the aged alloy in all conditions, the highest rate of crack growth was obtained for the lowest potential (−1200 mV_SCE_), and with increasing ageing time the crack growth rate at K = 45 MPa √m decreased. This was because, with increasing negative potentials (and therefore higher overpotentials for hydrogen production), additional diffusive hydrogen was formed, which increased the crack growth rate. Fractography of the alloy aged for 0.5 h showed that the intergranular hydrogen environment-assisted cracking (HEAC) occurred at all potentials applied. In the case of the alloy aged for 5 h, intergranular cracking attributable to HEAC occurred at −1200 and −1100 mV_SCE_. However, this alloy tested at −1000 mV_SCE_ showed no HEAC. In the case of testing the alloy aged for 50 h, a mixed intergranular–transgranular HEAC fracture morphology was observed only when tested at −1200 mV_SCE_.

### 5.3. Aluminium Alloys

Aluminium has a face-centred cubic (FCC) crystal lattice, similar to austenitic steels, and very low density (2700 kg cm^−3^). Due to the low density of aluminium alloys, they play an important role in many applications, especially where the weight of construction is crucial, such as in transport, hydrogen energy and aerospace industries [21]. Pure aluminium is soft and has low strength. To increase strength, alloying elements such as copper, magnesium, silicon, manganese, zinc, zirconium, nickel, caesium, cobalt and iron are added. This increase in strength is mainly due to precipitation hardening. Therefore, when discussing the hydrogen uptake in aluminium alloys, the influence of precipitates of intermetallic phases should be considered.

The influence of the structure and types of structural defects in an Al–Cu–Mg alloy after different treatments on the hydrogen trap sites was investigated by Safyari et al. [45]. The Al–Cu–Mg alloy was subjected to three different treatments: annealing at 495 °C for 1 h and quenched in water, so-called post-annealing rolling to reduce the thickness by 20%, and ageing at 190 °C for 9 h, which resulted in different densities of dislocations, vacancies and precipitates. Hydrogen was introduced during the SSRT test at a strain rate of 1.67 × 10^−7^ s^−1^ at room temperature in humid air controlled to 90% relative humidity. In the case of the alloy after annealing, no dislocations and precipitations were observed in the local-area-adjacent GBs. The cold rolling treatment increased the dislocation density near the GBs. Ageing resulted in precipitating the S’ (Al_2_CuMg) phase in the GBs and inside the grains. The HE susceptibility (HES) indexes calculated as the reduction in elongation due to testing in the different environments were 1.6%, 21.2% and 0% for the alloy after annealing, cold working and ageing, respectively. These results of the reduction in elongation can be considered comparable to those obtained for the 316 steel [34]. This means that the significantly lower strength properties of the aluminium alloy compared with 316 steel did not contribute to a significant decrease in HE resistance (HER).

**Figure 11:**
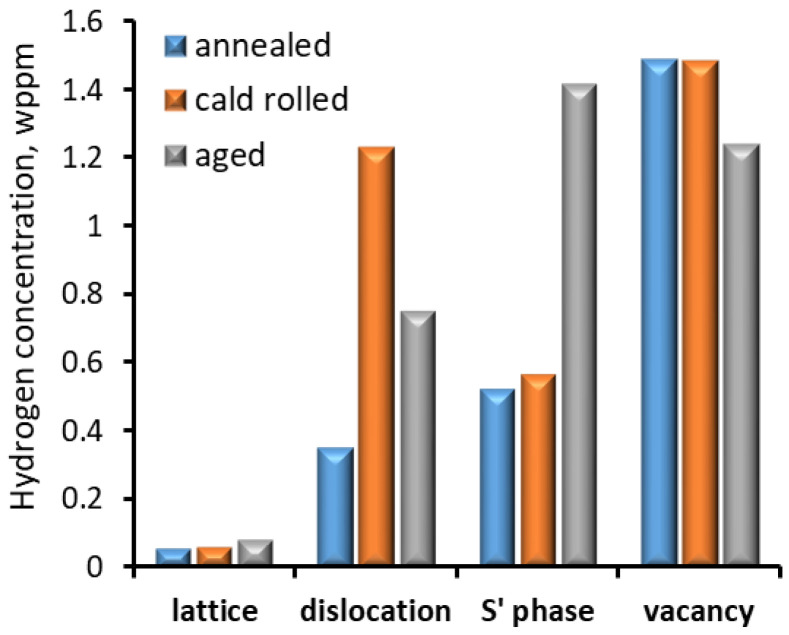
The hydrogen content in different trap side types in the Al–Cu–Mg alloy, based on data from [45].

The results obtained by Safyari et al. [45] indicate that mechanical treatment (cold rolling) significantly reduced the HER, in contrast to heat treatment (ageing) which had no effect on it. Thus, the increase in dislocation density along GBs due to cold rolling increased HE, in contrast to ageing which was characterised by a significantly lower hydrogen content on dislocations in GBs (Figure 11). Furthermore, the high hydrogen content on precipitates had little effect on the reduction in elongation. Thermal desorption spectroscopy (TDS) at a heating rate of 200 °C h^−1^ showed that, in addition to dislocations, vacancies, the S’ phase and the crystallographic lattice were the other sites of hydrogen trapping. The hydrogen concentration at different trap sites is shown in Figure 10. The most hydrogen was trapped in vacancies and the least in the crystallographic lattice. The effect of vacancies decreased in the case of the aged alloy, as the S’ phase precipitation contributed to more hydrogen being trapped there. Additionally, in the case of dislocations, more hydrogen was trapped in the alloy after cold rolling, where there was a high density of dislocations. This shows that the effect of individual hydrogen trapping sites was dependent on their density in the alloy structure. Considering that ageing and second-phase precipitation are a natural process of Al–Cu–Mg alloys, these results show the high resistance of such alloys to HE. On the other hand, as cold rolling is used for increasing endurance properties (mainly tensile strength), aluminium alloys should then be artificially aged to reduce the risk of HE.

An indentation test to investigate the effect of hydrogen charging conditions on the mechanical and tribological properties of an aluminium alloy was used by Georgiou et al. [26]. The alloy tested was 5754 aluminium alloy (3.2 wt.% Mg, 0.5 wt.% Mn, 0.4 wt.% Si, 0.4 wt.% Fe, 0.1 wt.% Cu and Al balance). Hydrogen charging was performed by cathodic polarization in an aqueous solution of 3 M HCl without poison as a hydrogen recombination inhibitor at room temperature. Graphite electrodes were used as anodes. The current densities were in the range of 25 to 300 mA cm^−2^ for a charging time of 2 h. Due to the lack of poison, the uptake of hydrogen was limited. The hydrogen content in the surface of the hydrogen-charged aluminium alloy ranged from 0.2 to 1 at.% hydrogen depending on the cathodic current density. The indentation tests showed that the hardness increased from 0.94 GPa for the uncharged alloy to 1.17 GPa for the current density of 75 mA cm^−2^. Further increases in current density resulted in a smaller increase in hardness. In addition to hardness, the elastic modulus also increased. This caused the resistance to plastic deformation to decrease compared with the uncharged alloy. The smallest decrease was obtained for the alloy charged at a current density of 75 mA cm^−2^ (5.5%), whereas the largest decrease was obtained for charging at a current density of 150 mA cm^−2^ (17.9%). Despite the increase in hardness, the wear depth in sliding testing also increased compared with the uncharged alloy and the greatest increase in wear depth was for charging at a current density of 150 mA cm^−2^ (26.5%).

**Figure 12:**
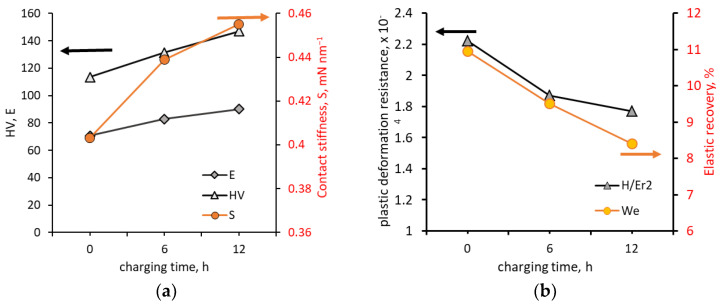
Effect of hydrogen charging on (**a**) hardness, elastic modulus and contact stiffness; (**b**) plastic deformation resistance and elastic recovery in aluminium alloy, based on data from [33].

Shin and Kim [33] also investigated the effect of hydrogen charging time on the mechanical properties of an aluminium alloy (0.82 wt.% Mg, 0.31 wt.% Si, 0.44 wt.% Fe, 0.18 wt.% Cr, 0.2 wt.% Cu, Al–balance). The hydrogen charging process was performed at a current density of 10 mA cm^−2^ in 2 N H_2_SO_4_ + 1 g L^−1^ Na_2_HAsO_4_·7H_2_O aqueous solution for 6 and 12 h. Surface analysis using a 3D microscope showed that, with increasing charging time, the surface roughness increased from Sa = 0.093 μm to Sa = 0.232 μm and this increase was due to the increasing number and size of hydrogen-induced pits. Tests using an indentation tester showed that the contact stiffness, hardness and reduced Young’s modulus increased but resistance to plastic deformation and elastic recovery decreased (Figure 12). Contact stiffness increased by 12.9% (from 0.403 mN nm^−1^ to 0.455 mN nm^−1^), hardness increased by approximately 29.7% (from 1.11 GPa to 1.44 GPa) and Young’s modulus increased by approximately 27.5% (from 70.725 GPa to 90.15 GPa). Resistance to plastic deformation decreased from 10.9% for uncharged alloy to 8.4% for the alloy after 12 h of charging. Shin and Kim [33] showed that the change in mechanical properties followed the effect of hydrogen on dislocation movement according to the HELP model.

Takano [63] found that cathodic hydrogen charging of 7075 aluminium alloy (Al–Zn–Mg–Cu) in 1 N H_2_SO_4_ solution at current densities of 10 mA cm^−2^ and 100 mA cm^−2^ at 45 °C during the SSRT test at a strain rate of 10^−5^ s^−1^ reduced both the tensile stress and elongation. The tensile stress decreased by 28% and 34%, respectively compared with the uncharged alloy, whereas the elongation decreased by 57% regardless of the current density. Thus, the greatest decrease was for the current density of 10 mA cm^−2^, while further increases in current density did not cause much change.

### 5.4. Titanium Alloys

Titanium and its alloys have a lower density than steel but a higher density than aluminium. Their very good strength properties and resistance to elevated temperatures and aggressive environments have led to its widespread application in many branches of industries. Unlike austenitic steel, nickel and aluminium, titanium has a hexagonal close-packed (HCP) crystal lattice. However, Lynch [5] showed that the hydrogen diffusion coefficients for Ti with HCP structure is comparable to Ni with FCC structure. The main alloying elements are aluminium and vanadium. Depending on the structure, titanium alloys are divided into three main groups: α-phase and near α-phase alloys, e.g., Ti–6Al–2Sn–4Zr–2Mo–0.1Si alloy [41]; α + β-phase alloys, e.g., Ti–6Al–4V alloy [64], Ti–5Al–5Mo–2V–2Cr–2Sn–2Zr alloy [65]; and β-phase alloys, e.g., Ti–3Al–8V–6Cr–4Zr–4Mo alloy [66].

The effect of hydrogen content on the properties and fracture of α-phase and near α-phase alloys has been investigated for Ti–6Al–2.8Sn–4Zr–0.5Mo–0.4Si–0.1Y alloy (Ti600) [40] and Ti–6Al–2Sn–4Zr–2Mo–0.1Si alloy [41], which are near-α titanium alloys. However, thermal or thermo-mechanical treatments can cause the formation of a certain amount of β-phase. The Ti–6Al–2.8Sn–4Zr–0.5Mo–0.4Si–0.1Y alloy was charged with hydrogen using a furnace at 750 °C and pressure of 5 × 10^−4^ Pa, into which hydrogen was introduced (1 L min^−1^) [30]. This treatment resulted in martensitic transformation β − Ti phase → α″ phase and eutectoid transformation β_H_ → δ + α phases. Zhang et al. [40] found that, as the hydrogen content increased, the β-phase content increased and the martensitic transformation temperature decreased. Hydrogen improved the stability of the β phase, reduced the critical cooling rate, which is important in many applications, and also reduced the characteristic temperature of martensitic transformation. Examination of the elongation and yield stress of the Ti600 alloy was carried out at temperatures between 840 °C and 960 °C and a strain rate of 5 × 10^−4^ s^−1^ [40]. The elongation of the alloy without hydrogen charging increased with temperature from 230% to 275%. Hydrogen charging contributed to an even higher elongation. At 860 °C, regardless of the hydrogen content, elongation was greater than 260%. The maximum improvement (300%) was achieved by the alloy with a hydrogen content of 0.2 wt.%. Further increase in temperature caused a decrease in elongation improvement. Furthermore, the elongation of the hydrogenated alloy did not change monotonically with temperature, but first increased and then decreased. With increasing temperature, the hydrogen content increased in the alloy where the elongation was lower than in the unsaturated alloy. The exception was the alloy with a hydrogen concentration of 0.5 wt.%, whose elongation increased with temperature. The elongation of this alloy reached a maximum value of 335% at 960 °C. Hydrogen also reduced the yield stress compared with the unloaded alloy.

**Figure 13:**
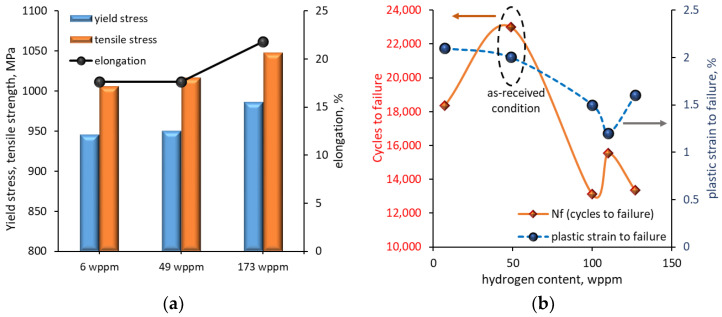
Effect of hydrogen content on (**a**) yield stress, tensile strength and elongation; (**b**) number of cycles to failure and plastic strain to failure in Ti–6Al–2Sn–4Zr–2Mo–0.1Si alloy, based on data from [41].

The Ti–6Al–2Sn–4Zr–2Mo–0.1Si alloy was subjected to a thermo-mechanical treatment consisting of forging at 28 °C to ~58% strain, solution heat treatment at 56 °C and air cooling, followed by ageing at 593 °C for 8 h and ageing while air cooled to room temperature [41]. This treatment resulted in a bimodal microstructure consisting of globular primary α-phase grains (~65 to 70 vol%) and lamellar transformed β-phase regions (~30 to 35 vol%). The Ti–6Al–2Sn–4Zr–2Mo–0.1Si alloy was then charged with hydrogen at a temperature of 550–600 °C using the Sievert apparatus [41]. In order to investigate the effect of hydrogen on mechanical properties, the tensile tests were conducted at room temperature and at a strain rate of 1 × 10^−4^ s^−1^. In addition, fatigue tests were carried out at room temperature, with a loading ratio R = 0, frequency of 0.5 Hz and a maximum stress of 0.95 × yield stress. Similar to the results in [40], an increase in hydrogen content increased elongation. An increase in yield stress and tensile strength was also achieved (Figure 13a). In contrast to the tensile properties, the fatigue tests showed that hydrogen charging reduced both the number of cycles to failure and the plastic strain to failure compared with the uncharged alloy (Figure 13b). Thus, despite the improvement in mechanical properties, fatigue life was reduced.

The most studied dual-phase (α + β) titanium alloy is the Ti–6Al–4V alloy. The effect of hydrogen on the cracking of this alloy was studied by Tal-Gutelmacher and Eliezer [64]. Lokoshenko et al. [42] investigated the creep and long-term strength of this alloy and Kolachev et al. [43] investigated the fatigue resistance. Similar to Sinha et al. [41] who tested near a-phase titanium alloys, Lokoshenko et al. [42] and Kolachev et al. [43] also used the thermodiffusion method to saturate the Ti–6Al–4V alloy with hydrogen. Creep tests showed that, with increasing hydrogen content and test stress, the steady-state creep rate and threshold creep strain decreased, while the time to fracture increased [42]. In the case of fatigue tests, the fatigue strength, taken as the stress obtained for 1 × 10^6^ cycles, initially increased with increasing hydrogen content but then decreased [43]. The hydrogen content threshold at which the best fatigue resistance was observed depended on the initial heat treatment of the alloy and the conditions of the fatigue tests. Regardless of many factors, the hydrogen content greater than 0.04% reduced fatigue stress and fatigue life. These results are in agreement with the studies of Sinha et al. [41].

**Figure 14:**
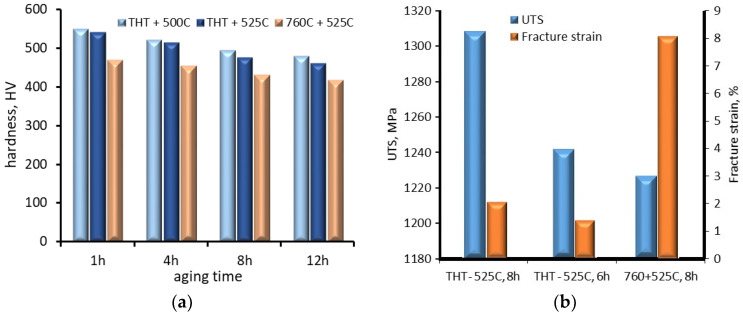
Effect of hydrogen and ageing time on (**a**) hardness and (**b**) UTS and failure strain, based on data from [67].

Examples of metastable β phase titanium alloys are Ti 10V–2Fe–3Al (Ti 10-2-3) and Ti–3Al–8V–6Cr–4Zr–4Mo. In the case of the Ti 10-2-3 alloy, the influence of hydrogen charging using thermo-hydrogen treatment (THT), where hydrogen is introduced during the heat treatment at temperatures between 550 °C and 795 °C, on phase stability and mechanical properties (microhardness, ultimate tensile strength and fracture strain) after ageing at different temperatures (500 °C and 525 °C) and times (1, 4, 8 and 12 h) has been investigated by Macin and Christ [67]. Initially, the alloy had a structure consisting of the β-phase (BCC Ti) and α_P_-phase (HCP Ti). Solution treatment (ST) at 760 °C for 1 h resulted in an increasing volume fraction of the α_P_ phase grains and the formation of the strengthening α_S_ phase. Ageing of the alloy at 525 °C for 8 h after ST resulted in an increase in the volume of the fraction of the α_S_ phase and the α_GB_ phase (the soft continuous layer at the grain boundaries of the β phase) and a reduction in the β phase grains to about 5 μm. The effect of hydrogen on the structure of the alloy depended on the hydrogen uptake value. At hydrogen intake values up to 32 at.%, hydrogen was mainly dissolved in the β phase. This was due to the fact that hydrogen is a β-phase-stabilizing alloying element. Consequently, the alloy consisted of α and β phases. When the hydrogen content exceeded 32 at.%, regions with two-phase (β + hydride) and three-phase (β + α + hydride) structures were formed. It should be noted that the hydrides formed in these structures differed in chemical composition, stoichiometry and crystal structure. The hydride content increased with increasing hydrogen content in the alloy. Ageing of the alloy reduced the volume fraction of the α_S_ phase, and increased α_GB_-phased layers and the size of α_GB_-phase precipitates. Hardness measurements showed that hydrogen increased the hardness of the alloy, but ageing decreased it. The decrease in hardness was dependent on the ageing temperature and time (Figure 14a). For example, when the ageing temperature was increased from 500 °C to 525 °C and the ageing time was 1 h, the hardness decreased by only 10 HV. In the case of ageing at 525 °C for 12 h, the decrease in hardness compared with ageing for 1 h was 80 HV. In comparison, ageing the alloy at 760 °C for 12 h after ST resulted in a reduction in hardness of 52 HV. In contrast to hardness, UTS and elongation at break (fracture strain) increased with increasing ageing time (Figure 14b). Comparing the UTS and fracture strain of the alloy after ST at 760 °C and ageing for 8 h with that of the alloy after hydrogen charging and ageing for 8 h, there is a significant decrease in UTS and an increase in fracture strain.

The Ti–3Al–8V–6Cr–4Zr–4Mo alloy after hydrogen charging at constant pressure for 8 h at 350 °C (623 K) and ageing at 482 °C for 28 h was investigated using TEM, XRD and SSRT tests [66]. The as-received alloy in the quenched condition had β-phase grains with an average diameter of 26 μm. Vacuum ageing for 28 h at 482 °C (755K) followed by air cooling resulted in the formation of HCP α-phase precipitates in a BCC β-phase matrix. The volume fraction of the α-phase was approximately 34%. Hydrogen charging to a content of 38 at.% did not change the phase composition and the alloy consisted only of α- and β-phases. Further increases of hydrogen content in the alloy resulted in the appearance of FCC δ-hydrides. The FCC δ-hydrides were formed in the HCP α-phase precipitates, while hydrogen remained in solid solution in the β-phase at its maximum concentration. As the hydrogen content increased, the lattice parameter of the β-phase also increased, but for hydrogen contents up to about 20 at.%, the lattice parameter was lower than the initial value in the alloy in the as-received condition. Furthermore, the lattice parameter increased rapidly for hydrogen contents greater than about 35 at.%. SSRT tests showed that, for hydrogen contents up to about 3.5 at.%, elongation (failure strain) was in the range of 15–20%. Further increases in hydrogen content rapidly reduced the elongation to about 3%. Thus, the embrittlement problem affected the alloy at hydrogen contents above 3.5 at.%.

## 6. Summary

The paper presents the main aspects of the problem of hydrogen-induced material degradation, including the presentation of hydrogen saturation methods and a discussion of the phenomenon of hydrogen penetration into the material. When presenting hydrogen charging methods, special attention was paid to two main hydrogen saturation methods, i.e., the electrochemical method, in which the charged material is the cathode and the method of charging with hydrogen under high pressure. It has been shown that the most commonly used electrolytes are H_2_SO_4_, NaOH and NaCl. The current density ranged from 0.02 mA cm^−2^ to 40 mA cm^−2^, but was higher than 10 mA cm^−2^ in most of the investigations. The most common charging time was 24 h. A method of measuring the hydrogen diffusion coefficient was also presented. It was shown that this coefficient depends on the phase structure and grain size of the tested material. However, it was pointed out that there are also discrepancies in the values of this coefficient, and that the reasons for this are not yet known despite many studies. The most important models of hydrogen-induced material degradation and the main assumptions of basic models were presented. The influence of hydrogen presence on the destruction of materials such as steels, aluminium alloys, nickel alloys and titanium alloys, i.e., metallic materials used in devices in contact with hydrogen, was discussed. It has been shown that in the case of steel, the reduction in elongation can be 50% or even 67%. However, most studies have shown a lower reduction in elongation. Nickel alloys have significantly lower hydrogen diffusion coefficients than austenitic steels. However, the reduction in elongation observed was much greater than for austenitic steels, up to 87%. Does this mean that nickel alloys have a lower HER than austenitic steels? It is difficult to answer this question, as there are no studies in which austenitic steels and nickel alloys have been charged to the same hydrogen content and tested for HER. In addition, even a small difference in alloy composition, such as in the case of 304 and 316 steels and in 718 and 725 nickel alloys, can result in a large difference in HE resistance, as has been shown in this review. Aluminium alloys have an FCC structure similar to that of austenitic steels, but lower strength properties. However, the reduction in elongation reported was comparable to that of 316 steel. In addition, the study shows that dislocation density affects HE, in contrast to precipitates resulting from ageing, which have no effect on HER. For titanium alloys, hydrogen charging contributed to an increase in elongation for Ti600 alloy tested at temperatures from 840 °C to 960 °C. The maximum increase in elongation was 300%, which was achieved for the Ti600 alloy with a hydrogen content of 0.2 wt.% and tested at 860 °C. In the case of the Ti–6Al–2Sn–4Zr–2Mo–0.1Si alloy, hydrogen charging had little effect on yield strength, tensile strength and elongation. These cases suggest that titanium alloys are the most resistant to HE. However, it should be remembered that resistance to HE depends on many factors, as this work has attempted to show. This review has shown that HE resistance is dependent on grain size, the phase structure of the material, the type of treatment carried out prior to HE resistance testing, the type and conditions of these HE tests and the hydrogen content of the material. Due to the large number of factors influencing the result, the change in properties under hydrogen atmosphere conditions makes testing necessary all the time.

## Figures and Tables

**Table 1:** The examples of electrolyte solutions, current densities and charging times for hydrogen charging.

Tested Material	Electrolyte Solution	Current Density [mA cm^−2^]	Charging Time [h]	Ref.
Aluminium alloy	2 N H_2_SO_4_ + 1 g L^−1^ Na_2_HAsO_4_•7H_2_O	10	6, 12	[33]
316L steel	0.5 mol L^−1^ H_2_SO_4_ +	20	96	[34]
316L steel	1 g L^−1^ sodium pyrophosphate tetrabasic decahydrate	100	24	[29]
316L steel	1N H_2_SO_4_ +	20, 100	24, 48, 72	[30]
304 steel	0.25 g L^−1^ As_2_O_3_	0.1	24	[31]
304L steel	0.1 M NaOH + 0.3 wt.% NH_4_SCN	20, 100	24, 48, 72	[30]
Fe–19Cr–8Ni–0.14C steel	3.0% NaCl + 3.0% wt.% NH_4_SCN	3	-	[16]
X100 pipeline steel	0.1 M NaOH + 0.3 wt.% NH_4_SCN	10	6	[10]
API Grade 60 steel	3% NaCl + 3 g L^−1^ NH_4_SCN	0.5	3, 12, 24	[28]
API 5L X52 steel	5 wt.% NaCl (pH = 2.4)	0.05–1.0	120	[35]
API 5L X65 steel	3% NaCl + 0.3% NH_4_SCN	20	24	[32]
Fe–0.22C–1.40Si–1.80 Mn (wt.%) steel	H_2_SO_4_ (pH 1) + 10 g L^−1^ (NH_2_)_2_CS	30	1 min, 5 min	[27]
low-alloy high-strength steel	0.2M H_2_SO_4_ + 3 g L^−1^ NH_4_SCN dissolved in distilled water.	0–7.5	10 min	[36]
A516 grade 70 steel	0.5 M H_2_SO_4_ + 1 g L^−1^ CSN_2_H_4_	1, 10	24, 48, 72	[30]
9840 steel	1 N H_2_SO_4_ + 0.05 g L^−1^ NaAsO_3_	5, 8, 12	16.7	[13]
4340 steel	3.5 wt.% NaCl + 0.3 wt.% NH_4_SCN	14.29	6	[37]
Ni-based alloys	0.1 N H_2_SO_4_	15	18	[11]
